# Association of Habitual Physical Activity With Home Blood Pressure in the Electronic Framingham Heart Study (eFHS): Cross-sectional Study

**DOI:** 10.2196/25591

**Published:** 2021-06-24

**Authors:** Mayank Sardana, Honghuang Lin, Yuankai Zhang, Chunyu Liu, Ludovic Trinquart, Emelia J Benjamin, Emily S Manders, Kelsey Fusco, Jelena Kornej, Michael M Hammond, Nicole Spartano, Chathurangi H Pathiravasan, Vik Kheterpal, Christopher Nowak, Belinda Borrelli, Joanne M Murabito, David D McManus

**Affiliations:** 1 Department of Medicine, Division of Cardiology University of California San Francisco San Francisco, CA United States; 2 Department of Medicine Boston University School of Medicine Boston, MA United States; 3 Boston University School of Public Health Boston, MA United States; 4 Framingham Heart Study Framingham, MA United States; 5 Care Evolution Ann Arbor, MI United States; 6 Henry M Goldman School of Dental Medicine Center for Behavioral Science Research Boston University Boston, MA United States; 7 Department of Medicine UMass Medical School Worcester, MA United States

**Keywords:** hypertension, primary prevention, eCohort, physical activity, smartwatch, Apple Watch, home blood pressure

## Abstract

**Background:**

When studied in community-based samples, the association of physical activity with blood pressure (BP) remains controversial and is perhaps dependent on the intensity of physical activity. Prior studies have not explored the association of smartwatch-measured physical activity with home BP.

**Objective:**

We aimed to study the association of habitual physical activity with home BP.

**Methods:**

Consenting electronic Framingham Heart Study (eFHS) participants were provided with a study smartwatch (Apple Watch Series 0) and Bluetooth-enabled home BP cuff. Participants were instructed to wear the watch daily and transmit BP values weekly. We measured habitual physical activity as the average daily step count determined by the smartwatch. We estimated the cross-sectional association between physical activity and average home BP using linear mixed effects models adjusting for age, sex, wear time, antihypertensive drug use, and familial structure.

**Results:**

We studied 660 eFHS participants (mean age 53 years, SD 9 years; 387 [58.6%] women; 602 [91.2%] White) who wore the smartwatch 5 or more hours per day for 30 or more days and transmitted three or more BP readings. The mean daily step count was 7595 (SD 2718). The mean home systolic and diastolic BP (mmHg) were 122 (SD 12) and 76 (SD 8). Every 1000 increase in the step count was associated with a 0.49 mmHg lower home systolic BP (*P*=.004) and 0.36 mmHg lower home diastolic BP (*P*=.003). The association, however, was attenuated and became statistically nonsignificant with further adjustment for BMI.

**Conclusions:**

In this community-based sample of adults, higher daily habitual physical activity measured by a smartwatch was associated with a moderate, but statistically significant, reduction in home BP. Differences in BMI among study participants accounted for the majority of the observed association.

## Introduction

Understanding the relationship between physical activity and blood pressure (BP) is crucial because promoting physical activity might help address the community burden of hypertension. Several observational and interventional studies have explored the association between physical activity and BP. Data from community-based observational studies suggest that higher self-reported moderate to vigorous physical activity, but not overall physical activity, is associated with lower research center/in-office BP and lower prevalence of incident hypertension [1-6]. Similarly, in interventional studies, promoting moderate to vigorous physical activity leads to a consistent reduction in BP in both normotensive and hypertensive individuals [7,8]. Most individuals, however, spend the majority of their time performing light activities, such as walking, which are suboptimally quantified using physical activity questionnaires [9,10], but can be accurately measured using accelerometers or smartwatches. Understanding the relationship between habitual physical activity and BP might yield fruitful targets to address the community burden of hypertension.

Smartwatches are commercially available devices for monitoring habitual physical activity and can enhance phenotyping of community-dwelling individuals. Daily step count reported by smartwatches could be a useful measure of overall physical activity capturing all intensities of exercise. Home BP, when compared to in-office BP, is a stronger predictor of adverse cardiovascular outcomes and mortality [11]. Merging the vast data continuously being collected using smart gadgets, such as smartwatches and Bluetooth-enabled BP cuffs, not only enriches the phenotypical information for individuals, but also opens up the possibility of studying the relationship of these novel ambulatory phenotypes (eg, daily step count) with cardiovascular risk factors (such as home BP).

We hypothesized that higher habitual physical activity is associated with lower home BP. To that end, we leveraged data from the ongoing electronic Framingham Heart Study (eFHS) cohort [12] to assess the association between home BP and habitual physical activity measured using a smartwatch (daily step count). Additionally, we sought to study the moderators of this association by adjusting for previously known correlates of hypertension and physical activity.

## Methods

### Overview

The Framingham Heart Study (FHS) is a multigenerational cohort study that was originally designed to study the risk factors for cardiovascular disease [13]. The eFHS cohort started enrolling participants from the FHS Third Generation Cohort (Gen 3), multiethnic Omni Group 2 Cohort (Omni 2), and New Offspring Spouse Cohort in June 2016 during regular research center examination [12]. The participants were offered a smartphone app, a Nokia Withings BP cuff for home BP monitoring, and a smartwatch (Apple Watch, Series 0, started November 2016). To be eligible for the BP cuff and Apple Watch, the participants were required to own an iPhone with a compatible iOS version (version 9 or higher). We chose the Withings wireless BP device because it is Food and Drug Administration approved for home BP monitoring and it has been validated (mean differences between the device and mercury readings for systolic and diastolic BP of −0.2 [SD 5.0] mmHg and 0.4 [SD 4.2] mmHg, respectively) [14,15]. The research protocol was approved by the Boston University Medical Center Institutional Review Board. All participants provided written informed consent.

### Study Protocol

**Figure 1:**
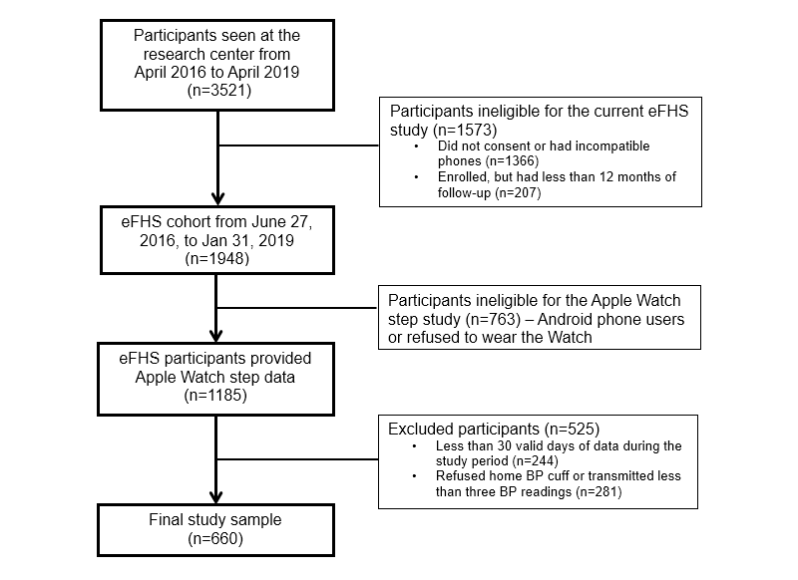
Cohort development diagram for the study. BP: blood pressure; eFHS: electronic Framingham Heart Study.

In this study, eFHS participants enrolled between June 27, 2016, and January 31, 2019, were included, and the median follow-up was 375 days from the date of enrollment (Figure 1). The participants were asked to wear the Apple Watch every day and transmit home BP readings every week. Habitual physical activity was measured as the average daily step count transmitted by the smartwatch. To reduce the bias related to low watch wear time, we only averaged the data from “valid days,” which were defined as those days with at least 5 hours of wear time. We, however, also performed sensitivity analyses leveraging an alternate threshold of 10 hours/day to define valid days. Of note, wear time from the Apple Watch was determined based on a combination of the heart rate and step count data transmitted by the watch. At rest, the watch measures the heart rate opportunistically every 5 minutes (heart rate is measured at a higher frequency during workouts) [16]. Therefore, any clock hours with more than one heart rate recording were included in the wear time. For the hours with one or less heart rate recordings, we only included clock hours with 30 or more steps in the wear time. Additionally, participants with less than 30 valid days were excluded (n=244; Figure 1). Several prior studies have measured physical activity by deploying accelerometers over short periods of time (eg, 1 week), which carries the risk of potential bias, as the participants might modify their behavior for the duration of the study [7,17]. In comparison to accelerometer-based studies, we leveraged physical activity measured by the smartwatch over a median follow-up of just over a year and did not include the data from participants with less than 30 valid days. Therefore, we likely captured the “habitual” level of physical activity, that is, the level of physical activity a participant will automatically perform if not being monitored (eg, using accelerometers).

### Home BP Measurement

The participants were advised to measure home BP once a week, ideally on the same day every week and at the same time during the day. They were advised to sit in a comfortable position with the feet flat on the floor and the left arm resting on a table with the palm up, rest for 5 minutes without talking, and then take the BP measurement. Participants were advised against BP measurement after exercise, after consuming caffeinated beverages, or after a high excitement activity. All BP recordings were date and time stamped. The BP recordings taken by the participants during the study period were averaged to calculate average home systolic and diastolic BP for each participant. We only included participants who transmitted three or more home BP recordings. We chose a threshold of three readings because prior studies have reported that home BP readings averaged over 3 days versus 7 or 10 days yield similar results [18,19]. We, however, did perform sensitivity analyses leveraging an alternate threshold of nine or more home BP readings (as described below). The variability in home BP readings was measured using the coefficient of variation (standard deviation/mean).

### Other Variables

Clinical and laboratory variables were measured during the examination at the research center [13]. Hypertension was defined as systolic BP ≥140 mmHg, diastolic BP ≥90 mmHg, and/or self-reported use of antihypertensive medications. Diabetes was defined as fasting plasma glucose ≥126 mg/dL and/or self-reported use of medications for diabetes. BMI was calculated by dividing body weight (in kg) by height (in meters) square.

### Statistical Methods

Baseline variables (from research center examination) were presented as mean (SD) or median (IQR) for continuous variables and as frequency (proportion) for categorical variables. The correlation between average daily step count and home systolic and diastolic BP was determined using the Spearman correlation coefficient and depicted using a correlation matrix. The association between average daily step count and home BP was measured using separate linear mixed effect regression models for systolic and diastolic BP. Primary models were adjusted for age, sex, familial structure/relatedness in the FHS, antihypertensive drug use, and watch wear time. The secondary model was further adjusted for BMI. We checked for interaction with wear time using the interaction term step count×wear time. For each model, we also performed sex-stratified analyses and tested assumption of linearity by inspecting the residual plots. As the FHS is a multigenerational study, to account for relatedness between the participants, we included the familial relatedness variable. This variable was derived from the self-reported pedigree structure by R package kinship and was treated as a random effect in our regression models. Additionally, sensitivity analyses were performed leveraging alternate inclusion thresholds for valid days (60 days or 90 days instead of 30 days), wear time (10 hours per valid day vs 5 hours per valid day), and home BP recordings (minimum nine home BP recordings vs three recordings). Similarly, we studied the association between home BP variability and average daily step count in primary and secondary models. Additionally, BMI-stratified exploratory analyses were performed to study the association between home BP and daily step count across various BMI strata (normal BMI <25 kg/m^2^, overweight BMI 25-29.9 kg/m^2^, and obese BMI ≥30 kg/m^2^). These stratified models were adjusted for age, sex, familial structure, antihypertensive drug use, and watch wear time (similar to the primary models). We also performed exploratory analyses using regression models 1 and 2 in a subsample of participants with a history of hypertension. To account for multiple comparisons in these exploratory post-hoc stratified analyses, Bonferroni correction for the *P* value was used to determine statistical significance (*P*<.05/3=.017). For other a priori models, the significant association was defined by a two-sided *P* value <.05. Box plots were used to show the distribution of average daily step count and home BP across BMI strata. All statistical analyses were performed using R software package version 3.5.0 (The R Project for Statistical Computing).

## Results

Our study sample consisted of 660 adults (mean age 53 years, SD 9 years; 387 [58.6%] women; 602 [91.2%] White; Table 1). Approximately one in five participants reported taking antihypertensive medications. The mean BMI was 27.8 kg/m^2^ (SD 5 kg/m^2^), and over two-thirds of participants were overweight (n=277) or obese (n=183). The baseline prevalence of smoking, diabetes, and cardiovascular disease was low. In Multimedia Appendix 1, we have presented the baseline characteristics of the study participants including all research center exam attendees and eFHS cohort participants. The eFHS participants were younger and more likely to be female, compared to all research center examinees. Other baseline characteristics were comparable between all eFHS participants and the study participants.

**Table 1:** Baseline characteristics of the study participants (N=660).

Variable			Value, mean (SD) or n (%)
Age (years), mean (SD)			53 (9)
Female sex, n (%)			387 (58.6%)
BMI (kg/m^2^), mean (SD)			27.8 (5.0)
Systolic BP^a^ (research center) (mmHg), mean (SD)			119 (14)
Diastolic BP (research center) (mmHg), mean (SD)			76 (9)
Antihypertensive drug use, n (%)			145 (22.0%)
Current smoking, n (%)			28 (4.2%)
Diabetes mellitus, n (%)			41 (6.2%)
**Race, n (%)**			
	White	602 (91.2%)	
	Black	14 (2.1%)	
	Hispanic	17 (2.6%)	
	Asian	13 (2.0%)	
	Other	14 (2.1%)	
Cardiovascular disease, n (%)			26 (3.9%)
Systolic BP (home) (mmHg), mean (SD)			122 (12)
Diastolic BP (home) (mmHg), mean (SD)			76 (8)
Daily step count, mean (SD)			7595 (2718)

^a^BP: blood pressure.

**Figure 2:**
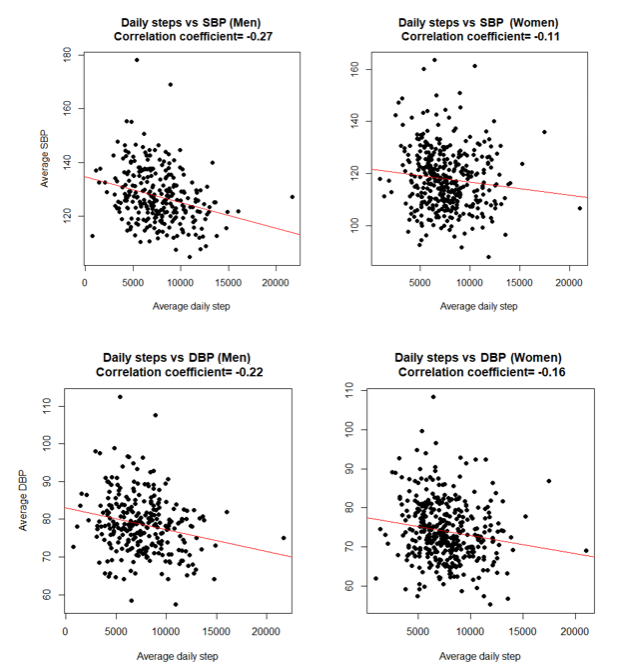
Scatter plots depicting the correlation of average daily step count with average home systolic and diastolic blood pressure. DBP: diastolic blood pressure; SBP: systolic blood pressure.

Over a median of 375 (25%-75%: 180-581) follow-up days, participants wore the Apple Watch for a median of 13.7 (IQR 12.4-14.8) hours per day and sent BP readings for 28 (IQR 11-63) weeks. The mean daily step count was 7595 (SD 2718). The mean home systolic and diastolic BP (mmHg) values were 122 (SD 12) and 76 (SD 8), respectively. In sex-stratified analyses, average daily step count was inversely correlated with home systolic and diastolic BP (in both men and women; Figure 2), that is, participants with a higher daily step count had lower home systolic and diastolic BP.

**Figure 3:**
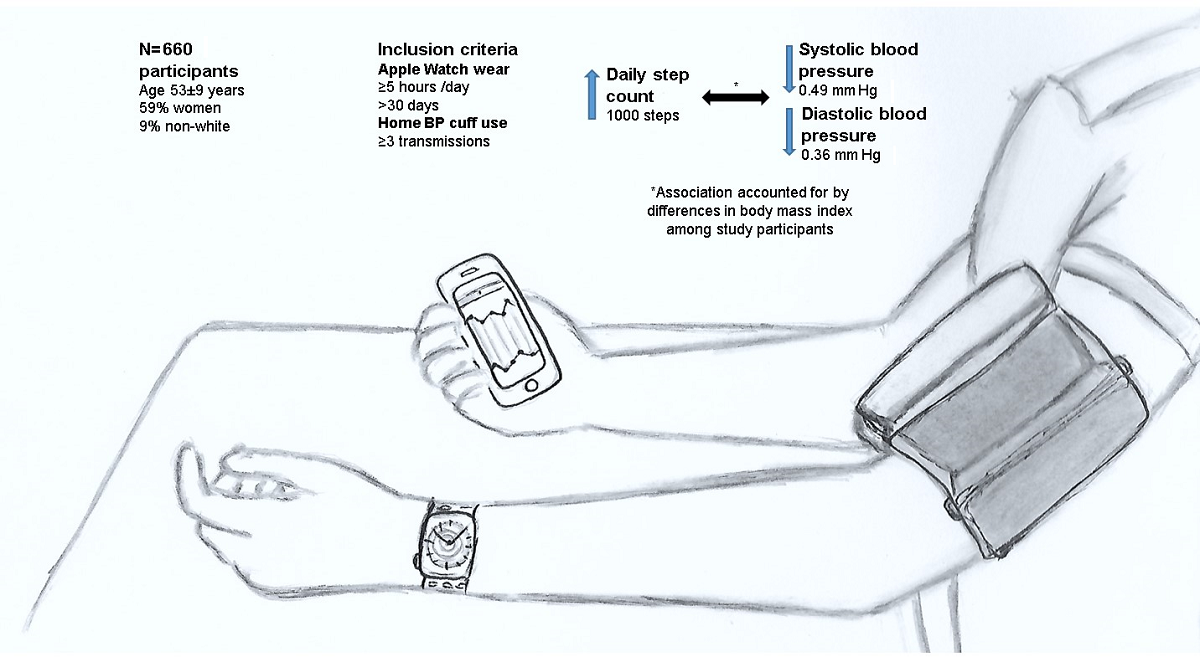
Association of daily step count with home blood pressure. BP: blood pressure.

In the regression models adjusted for age, sex, watch wear time, family structure, and antihypertensive drug use (model 1), each 1000-step increment was associated with a 0.49 mmHg lower home systolic BP and 0.36 mmHg lower home diastolic BP (Table 2, Figure 3). No significant interaction with wear time was noted (*P*=.82 for systolic BP analyses and *P*=.35 for diastolic BP analyses). In sex-stratified analyses, the findings were overall similar in men and women, although the *P* value did not reach the statistical significance threshold in men for diastolic BP. Given the same directionality of the association in men as the overall sample and subsample with women and a relatively weak correlation between diastolic BP and daily steps (correlation coefficient −0.22), we suspected that we were underpowered in our sex-stratified analyses. We have included the power calculation in Multimedia Appendix 2. With further adjustment for BMI (model 2), the strength of the association between daily step count and home BP attenuated and became statistically nonsignificant. When we studied the association of log-transformed daily step count with home BP, the results remained unchanged (Multimedia Appendix 3).

**Table 2:** Association of daily step count with home blood pressure using separate mixed linear effect models for systolic and diastolic blood pressure.

Home BP^a^ and participants		Model 1^b^				Model 2^c^		
		β^d^ (mmHg)	SE	*P* value	β^d^ (mmHg)		SE	*P* value
**Systolic BP**								
	All participants (n=660)	−0.49	0.17	.004	0.006		0.16	.97
	Women (n=387)	−0.47	0.23	.05	0.08		0.20	.70
	Men (n=273)	−0.53	0.24	.03	−0.14		0.24	.55
**Diastolic BP**								
	All participants (n=660)	−0.36	0.12	.003	−0.04		0.12	.76
	Women (n=387)	−0.45	0.16	.01	−0.08		0.14	.59
	Men (n=273)	−0.26	0.19	.18	−0.01		0.19	.94

^a^BP: blood pressure.

^b^Model 1 was adjusted for age, sex, family structure, reported antihypertensive drug use, and watch wear time.

^c^Model 2 was adjusted for model 1 covariates and BMI.

^d^β represents the change in BP (mmHg) for every 1000 increase in daily steps.

Of note, our primary analyses were restricted to the participants who had at least 30 active days and transmitted three or more home BP readings. We performed four sensitivity analyses to test the generalizability of our findings and increase the validity of our results as follows: (1) participants with 60 or more active days (n=611; Multimedia Appendix 4), (2) participants with 90 or more active days (n=578; Multimedia Appendix 5), (3) participants with nine or more home BP readings (n=540; Multimedia Appendix 6), and (4) using a 10 hours per valid day threshold (n=634; Multimedia Appendix 7). The results in sensitivity analyses were similar to the primary analyses, that is, there was an inverse association between daily step count and home BP in model 1, but the association became nonsignificant in model 2.

We also studied the association of home BP variability (coefficient of variation) with the daily step count using separate mixed linear effect models for systolic BP and diastolic BP in models analogous to the primary analyses (model 1 adjusted for age, sex, watch wear time, antihypertensive drug use, and family structure, and model 2 further adjusted for BMI). In these models, a higher daily step count was associated with significantly lower diastolic BP variability (but not systolic BP variability; Table 3). In sex-stratified analyses, the findings were overall similar in men and women, although the *P* value did not reach the statistical significance threshold in women for diastolic BP variability. Given the same directionality of the association in women as the overall sample and subsample with men, we suspected that we were underpowered in our sex-stratified analyses.

To further explore the association of BMI with home BP and daily step count, we performed BMI-stratified analyses. In these models adjusted for age, sex, familial structure, antihypertensive drug use, and watch wear time, we did not observe any statistically significant association of home BP (separate models for systolic and diastolic BP) with daily step count (Table 4). In Multimedia Appendix 8 and Multimedia Appendix 9, we depict the distribution of average daily step count and home BP across different BMI strata (normal weight, overweight, and obese). There was a significant trend toward a lower step count and higher systolic and diastolic BP with a higher BMI stratum (*P*<.001). In Multimedia Appendix 10, we have shown scatter plots depicting the correlation of average daily step count and home BP stratified by different BMI categories. We also performed exploratory analyses among participants with a history of hypertension (n=183). Compared to participants without a history of hypertension, participants with a history of hypertension had a lower daily step count, were older, were more likely to be men, and had a higher BMI (Multimedia Appendix 11). In these analyses, we did not observe a significant association between step count and systolic or diastolic BP in models 1 and 2 (Multimedia Appendix 12). Although the findings might be because of underpowering, they suggest that the relationship of step count with home BP is not stronger among participants with a history of hypertension. When we treated home BP as a tertile variable, we observed results similar to those in our primary analyses (Multimedia Appendix 13).

**Table 3:** Association of daily step count with home blood pressure variability (coefficient of variation).

Home BP^a^ and participants		Model 1^b^				Model 2^c^		
		β^d^ (mmHg)	SE	*P* value	β^d^ (mmHg)		SE	*P* value
**Systolic BP**								
	All participants (n=660)	−0.0002	0.0003	.65	−0.00017		0.00034	.62
	Women (n=387)	0.0001	0.0004	.72	0.00018		0.00042	.67
	Men (n=273)	−0.0006	0.0005	.25	−0.00074		0.00057	.19
**Diastolic BP**								
	All participants (n=660)	−0.0011	0.0004	.003	−0.00095		0.00037	.01
	Women (n=387)	−0.0008	0.0004	.09	−0.00060		0.00045	.19
	Men (n=273)	−0.0016	0.0006	.01	−0.00161		0.00064	.01

^a^BP: blood pressure.

^b^Model 1 was adjusted for age, sex, family structure, reported antihypertensive drug use, and watch wear time.

^c^Model 2 was adjusted for model 1 covariates and BMI.

^d^β represents the change in BP (mmHg) for every 1000 increase in daily steps.

**Table 4:** Association of daily step count with home blood pressure in models stratified by BMI.

Home BP^a^ and participants			Normal (BMI <25 kg/m^2^)^b^ (n=200)							Overweight (25 ≤ BMI < 30 kg/m^2^)^b^ (n=277)							Obese (BMI ≥30 kg/m^2^)^b^ (n=183)				
			β^c^ (mmHg)		SE		*P* value		β^c^ (mmHg)			SE		*P* value		β^c^ (mmHg)			SE		*P* value
**Systolic BP**																					
	All participants	−0.04		0.25		.88		−0.34			0.23		.14		0.10			0.36		.79	
	Women	0.00		0.28		.99		−0.28			0.36		.43		0.03			0.58		.96	
	Men	−0.01		0.50		.98		−0.48			0.30		.11		0.19			0.45		.67	
**Diastolic BP**																					
	All participants	0.08		0.18		.66		−0.34			0.16		.04		−0.02			0.26		.95	
	Women	−0.01		0.20		.97		−0.37			0.24		.12		−0.37			0.42		.38	
	Men	0.38		0.45		.40		−0.33			0.23		.15		0.31			0.34		.36	

^a^BP: blood pressure.

^b^Models were adjusted for age, sex, family structure, reported antihypertensive drug use, and watch wear time.

^c^β represents the change in BP (mmHg) for every 1000 increase in steps.

## Discussion

### Principal Findings

In this study of community-dwelling participants, we measured habitual physical activity using a study smartwatch and home BP device. We observed an inverse association of higher habitual physical activity with lower home BP, even after adjusting for age, sex, watch wear time, antihypertensive drug use, and family structure. The association between physical activity and home BP, however, was rendered statistically nonsignificant after further adjusting for BMI, suggesting the mediating role of BMI.

When studied in community-based samples, the association of physical activity and BP remained controversial [3,20] and is perhaps dependent on the intensity of activity. For example, in a community-dwelling sample from the Dutch Lifelines cohort (n=125,402), higher self-reported commuting and leisure time moderate to vigorous physical activity was associated with lower BP in a dose-dependent manner (cross-sectional analysis) [1]. Similarly, in a sample of African American participants derived from the Jackson Heart Study (n=1311), lower baseline moderate to vigorous physical activity, but not overall physical activity, was associated with an increased risk of incident hypertension over a median follow-up of 8 years [2]. On the contrary, in 1717 participants of the Framingham Offspring study, the self-reported physical activity index (a composite score of daily physical activity and sedentary behavior) did not emerge as an independent predictor of incident hypertension in multivariable-adjusted models [6]. A similar lack of an independent association of physical activity with incident hypertension was observed in the participants of the National Health and Nutrition Examination Survey [5] and Coronary Artery Risk Development in (Young) Adults Study [4]. Since 2016, eFHS participants have undergone digital phenotyping using research smartwatches and BP cuffs, thereby enriching the existing information about these FHS participants [12]. With the goal of better understanding the relationship between physical activity and BP, in our current investigation, we studied the association of a common measure of physical activity (daily step count) [21] and home BP (a powerful predictor of adverse cardiovascular outcomes) [11]. The demographic-adjusted regression model (primary model) revealed an inverse association of habitual physical activity with home BP. However, with further adjustment for BMI (secondary model), the association became attenuated and nonsignificant, suggesting that BMI accounted for most or all of the association between habitual physical activity and home BP. The results of our exploratory BMI-stratified models further confirmed this observation as no significant association of habitual physical activity with home BP was observed in any BMI subgroups (normal weight, overweight, or obese).

Our findings, when taken together with prior observations, suggest that the effect of overall physical activity on BP in community-based settings is likely mediated via correlates of physical activity such as obesity. While several prior studies have reported an association of moderate to vigorous physical activity with BP and incident hypertension [1,2], we were unable to study the relationship of exercise intensity with home BP as the current wearables (including the Apple Watch used in our study) do not accurately discriminate between the intensity of exercise [22-24]. Nevertheless, community-dwelling individuals spend the majority of their time performing light physical activity (such as walking), and over the last decade, several studies have reported the beneficial effects of light physical activity on overall metabolic profile [25-27] and mortality [28]. It is plausible that light physical activity modulates BP via improvement in the metabolic profile (eg, BMI in our study) [29,30], whereas moderate to vigorous physical activity lowers BP more directly by reducing the vascular tone [3]. In fact, interventions aimed at increasing moderate to vigorous physical activity consistently lead to a reduction in BP in both normotensive and hypertensive individuals [7,8]. Of note, we observed an inverse association of home diastolic BP variability, an independent predictor of adverse cardiovascular outcomes [31], with higher step count independent of BMI. The association of habitual physical activity with reduced diastolic BP variability might be attributable to its effect on autonomic modulation [32]. Our findings suggest that overall habitual physical activity could lead to reduced cardiovascular risk despite the lack of an independent association with absolute home BP.

Another major implication of our study is that smartwatches provide scalable instruments to measure habitual physical activity in community-based settings. Prior studies have reported a significant reduction in BP-leveraging pedometer and smartphone-based physical activity promotion interventions [7]. Home BP is a stronger predictor of adverse cardiovascular outcomes compared with in-office BP [11], and self-monitoring of postexercise hypotension can improve exercise adherence [33]. Nearly one in five Americans currently own a smartwatch [34]. With the burgeoning field of wearable smart devices, our findings raise the possibility of leveraging smartwatches and home BP monitoring to promote physical activity to address the community burden of hypertension and associated comorbidities such as obesity [35].

### Limitations

Our study has several limitations. First, the participants in our study were predominantly White and of European ancestry, and had a higher average daily step count (7595 steps/day) than the US average (4774 steps/day) [36]. Further, we acknowledge that our study sample (n=660) represents a subset of younger participants who attended the research center examination (n=3521). Although the baseline characteristics of the eFHS cohort and our study sample were similarly distributed, the possibility of selection bias exists. Our findings should be validated in samples with older participants and in racially diverse samples as there is evidence supporting the differential association of physical activity with incident hypertension, based on race [37]. Second, the analyses performed in our observational study are cross-sectional in nature. Therefore, our findings do not imply a causal association between lower PA and higher BP. However, our eFHS cohort is embedded in the overall FHS, and as these participants are systematically followed over the next few years, we would be able to study the pattern of habitual physical activity that relates to a higher risk of incident hypertension. Third, although the association between habitual physical activity and home BP was statistically significant, whether a 0.49 mmHg lower systolic BP and 0.36 mmHg lower diastolic BP per 1000 daily steps translates to a clinically meaningful impact in the community should be assessed in future studies. Even a 2 mmHg reduction in the average diastolic BP, however, would lead to a 17% decrease in hypertension prevalence [38]. From the clinical standpoint, considering the large number of daily steps required to achieve a modest reduction in BP, our findings might not translate to significant benefits at the individual level. Fourth, we relied on outputs from the Apple Watch proprietary algorithms to measure physical activity in our study. Although validated for measuring physical activity, these algorithms are not available publicly [22,39].

### Conclusions

In this community-based sample of middle-aged adults who were enrolled in an electronic cohort at the time of their routine research center examination, we observed that higher habitual physical activity measured by a smartwatch was associated with a moderate, but statistically significant, reduction in home BP. Differences in BMI among study participants accounted for the majority of the observed association. The results of our study lay the ground work for leveraging smart devices to promote physical activity and improve the cardiometabolic phenotype in the community.

## Appendix

Multimedia Appendix 1 Characteristics of the study participants compared to the overall electronic Framingham Heart Study cohort and all research center attendees during the study enrollment period.

Multimedia Appendix 2 Additional methods and results.

Multimedia Appendix 3 Association of log-transformed daily step count with home blood pressure using separate mixed linear effect models for systolic and diastolic blood pressure.

Multimedia Appendix 4 Association of daily step count with home blood pressure in participants with 60 or more active days.

Multimedia Appendix 5 Association of daily step count with home blood pressure in participants with 90 or more active days.

Multimedia Appendix 6 Association of daily step count with home blood pressure in participants with nine or more blood pressure readings.

Multimedia Appendix 7 Association of daily step count with home blood pressure using a threshold of 10 hours/day to define active days.

Multimedia Appendix 8 Box plots depicting average daily step count stratified by BMI categories. N=200 for normal weight, N=277 for overweight, and N=183 for obese.

Multimedia Appendix 9 Box plots depicting average home systolic and diastolic blood pressure stratified by BMI categories. N=200 for normal weight, N=277 for overweight, and N=183 for obese. DBP: diastolic blood pressure; SBP: systolic blood pressure.

Multimedia Appendix 10 Scatter plots depicting the correlation between average daily step count and home blood pressure stratified by BMI categories.

Multimedia Appendix 11 Baseline characteristics of participants with and without a history of hypertension.

Multimedia Appendix 12 Association of daily step count with home blood pressure in participants with a history of hypertension.

Multimedia Appendix 13 Association of daily step count with home blood pressure tertile.

## Abbreviations

BP: blood pressure
eFHS: electronic Framingham Heart Study
FHS: Framingham Heart Study
