# Supplementary material for: Association of Habitual Physical Activity With Home Blood Pressure in the Electronic Framingham Heart Study (eFHS): Cross-sectional Study
Source: J Med Internet Res. 2021 Jun 24;23(6):e25591. doi: 10.2196/25591 (PMC8277303; doi:10.2196/25591)
Supplement: Multimedia Appendix 1 [file jmir_v23i6e25591_app1.docx]

**Multimedia Appendix 1.** Characteristics of the study participants compared to the overall electronic Framingham Heart Study cohort and all research center attendees during the study enrollment period.

| **Characteristics^+^** | **Research center attendees**  **(n=3521)** | **eFHS**  **cohort**  **(n=1948)** | **Study participants (n=660)** |
| --- | --- | --- | --- |
| **Age, years** | 55 ± 9 | 53 ± 9 | 53 ± 9 |
| **Female sex, n (%)** | 1896 (53.8) | 1109 (56.9) | 387 (58.6) |
| **Body mass index, kg/m^2^** | 28.6 ± 6.2 | 28.2 ± 5.6 | 27.8 ± 5.0 |
| **Systolic BP (research center), mm Hg** | 120 ± 14 | 118 ± 14 | 119 ± 14 |
| **Diastolic BP (research center), mm Hg** | 76 ± 9 | 76 ± 8 | 76 ± 9 |
| **Antihypertensive use, n (%)** | 867 (24.6) | 407 (20.9) | 145 (22.0) |
| **Current smoking, n (%)** | 234 (6.6) | 108 (5.5) | 28 (4.2) |
| **Diabetes mellitus, n (%)** | 310 (8.8) | 121 (6.2) | 41 (6.2) |
| **Race** |  |  |  |
| White | 3233 (91.8) | 1813 (93.1) | 602 (91.2) |
| Black | 59 (1.7) | 30 (1.5) | 14 (2.1) |
| Hispanic | 106 (3.0) | 45 (2.3) | 17 (2.6) |
| Asian | 71 (2.0) | 28 (1.4) | 13 (2.0) |
| Other | 52 (1.5) | 32 (1.6) | 14 (2.1) |
| **Cardiovascular disease, n (%)** | 164 (4.7) | 67 (3.4) | 26 (3.9) |

**^+^**Presented are mean ± standard deviation for continuous traits and n (%) for dichotomous traits.

*Study enrollment period extended from June 27, 2016 to January 31, 2019
