# Supplementary material for: Association of Habitual Physical Activity With Home Blood Pressure in the Electronic Framingham Heart Study (eFHS): Cross-sectional Study
Source: J Med Internet Res. 2021 Jun 24;23(6):e25591. doi: 10.2196/25591 (PMC8277303; doi:10.2196/25591)
Supplement: Multimedia Appendix 2 [file jmir_v23i6e25591_app2.docx]

**Multimedia Appendix 2.** Additional methods and results.

*Research center BP measurement and its association with habitual physical activity*

During the research center visit, the BP was measured twice by a trained research center physician or nurse practitioner using a standard protocol, and the average of the two readings was used. The correlation between the research center BP and home BP was depicted in correlation matrices for systolic and diastolic BP. The association between research center BP and average daily step count was studied using primary and secondary regression models, similar to the home BP analyses.

The average research center systolic and diastolic BP were 119±14 and 76±19 mm Hg. There was moderate correlation between the research center BP and home BP (correlation coefficient =0.66 and 0.59 for systolic and diastolic BP, **Figure S1**). We also studied the association of BP measured in the research center with daily step count. Unlike our primary analyses with home BP, no significant association was observed between the research center BP and daily step count (**Table S1**).

*Power calculation for sex-stratified analyses*

We performed power calculation for our model 1 of our primary analyses. To achieve 80% power with 237 men in our study, we needed a correlation coefficient of at least 0.17 to detect significant association between BP and step count. With variables such as age and wear time explaining some of the association between step count and BP, an even higher correlation coefficient was needed. Similarly, for the BP variability analyses, to achieve 80% with 237 men in our study, we needed a correlation coefficient with absolute value at least 0.17 to detect significant association between BP variability and step count. We observed a correlation coefficient of -010 for SBP variability (men=-0.14, women=-0.06) and -0.18 for DBP variability (men=-0.21, women=-0.16).

**Figure S1.** Scatter plots depicting the correlation between research center blood pressure and home blood pressure.


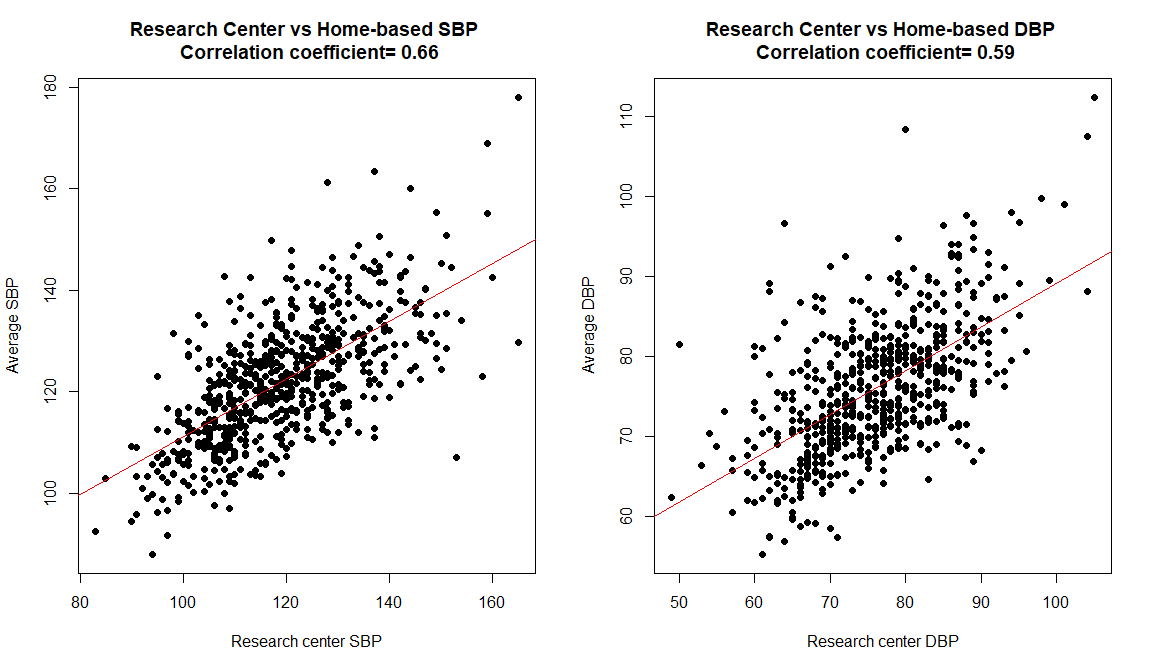


**Table S1.** Association of daily step count with research center blood pressure.

| Home BP | Participants | Model 1* | | | Model 2^†^ | | |
| --- | --- | --- | --- | --- | --- | --- | --- |
|  |  | β^‡^ (; mm Hg) | SE | P-value | β^‡^ (; mm Hg) | SE | P-value |
| Systolic BP | All participants  n=941^§^ | -0.05 | 0.17 | 0.79 | 0.282 | 0.17 | 0.10 |
|  | Women  n=567 | 0.02 | 0.21 | 0.94 | 0.36 | 0.22 | 0.11 |
|  | Men  n=374 | -0.14 | 0.26 | 0.59 | 0.13 | 0.27 | 0.64 |
| Diastolic BP | All participants  n=941 | 0.04 | 0.11 | 0.72 | 0.24 | 0.11 | 0.04 |
|  | Women  n=567 | -0.02 | 0.14 | 0.87 | 0.19 | 0.14 | 0.17 |
|  | Men  n=374 | 0.12 | 0.18 | 0.52 | 0.29 | 0.19 | 0.13 |

*Model 1 was adjusted for age, sex, family structure, reported antihypertensive agent use, and watch wear time

^†^Model 2 was adjusted for model 1 covariates and body mass index.

^‡^β represents the change in BP (mmHg) for every 1,000 increase in daily steps

^§^The sample for these analyses consisted of all participants who wore the smartwatch for ≥30 days (not just the participants who took home BP cuff)
