# Supplementary material for: Association of Habitual Physical Activity With Home Blood Pressure in the Electronic Framingham Heart Study (eFHS): Cross-sectional Study
Source: J Med Internet Res. 2021 Jun 24;23(6):e25591. doi: 10.2196/25591 (PMC8277303; doi:10.2196/25591)
Supplement: Multimedia Appendix 3 [file jmir_v23i6e25591_app3.docx]

**Multimedia Appendix 3.** Association of log-transformed daily step count with home blood pressure using separate mixed linear effect models for systolic and diastolic blood pressure.

| Home BP | Participants | Model 1* | | | Model 2^†^ | | |
| --- | --- | --- | --- | --- | --- | --- | --- |
|  |  | β^‡^ (; mm Hg) | SE | P-value | β^‡^ (; mm Hg) | SE | P-value |
| Systolic BP | All participants n=660 | -3.91 | 1.26 | 0.002 | 969.196 | 1.15 | 0.40 |
|  | Women  n= 387 | -3.94 | 1.79 | 0.03 | 1282.23 | 1.54 | 0.40 |
|  | Men  n= 273 | -3.13 | 1.70 | 0.07 | 770.51 | 1.68 | 0.65 |
| Diastolic BP | All participants n=660 | -2.53 | 0.90 | 0.005 | 638.537 | 0.85 | 0.45 |
|  | Women  n= 387 | -3.27 | 1.23 | 0.01 | 207.16 | 1.08 | 0.85 |
|  | Men  n= 273 | -1.27 | 1.31 | 0.33 | 1204.68 | 1.33 | 0.37 |

*Model 1 was adjusted for age, sex, family structure, reported antihypertensive agent use, and watch wear time

^†^Model 2 was adjusted for model 1 covariates and body mass index.

^‡^β represents the change in BP (mmHg) for every 1,000 increase in daily steps
