# Supplementary material for: Association of Habitual Physical Activity With Home Blood Pressure in the Electronic Framingham Heart Study (eFHS): Cross-sectional Study
Source: J Med Internet Res. 2021 Jun 24;23(6):e25591. doi: 10.2196/25591 (PMC8277303; doi:10.2196/25591)
Supplement: Multimedia Appendix 4 [file jmir_v23i6e25591_app4.docx]

**Multimedia Appendix 4.** Association of daily step count with home blood pressure in participants with 60 or more active days.

| Home BP | Participants | Model 1* | | | Model 2^†^ | | |
| --- | --- | --- | --- | --- | --- | --- | --- |
|  |  | β^‡^ (; mm Hg) | SE | P-value | β^‡^ (; mm Hg) | SE | P-value |
| Systolic BP | All participants  n=611 | -0.43 | 0.18 | 0.017 | 0.065 | 0.16 | 0.69 |
|  | Women  n=366 | -0.40 | 0.24 | 0.10 | 0.13 | 0.21 | 0.52 |
|  | Men  n=245 | -0.46 | 0.26 | 0.08 | -0.07 | 0.26 | 0.80 |
| Diastolic BP | All participants  n=611 | -0.34 | 0.13 | 0.009 | -0.01 | 0.12 | 0.96 |
|  | Women  n=366 | -0.42 | 0.16 | 0.01 | -0.06 | 0.14 | 0.65 |
|  | Men  n=245 | -0.21 | 0.20 | 0.31 | 0.04 | 0.21 | 0.83 |

*Model 1 was adjusted for age, sex, family structure, reported antihypertensive agent use, and watch wear time

^†^Model 2 was adjusted for model 1 covariates and body mass index.

^‡^β represents the change in BP (mmHg) for every 1,000 increase in daily steps
