# Supplementary material for: Association of Habitual Physical Activity With Home Blood Pressure in the Electronic Framingham Heart Study (eFHS): Cross-sectional Study
Source: J Med Internet Res. 2021 Jun 24;23(6):e25591. doi: 10.2196/25591 (PMC8277303; doi:10.2196/25591)
Supplement: Multimedia Appendix 5 [file jmir_v23i6e25591_app5.docx]

**Multimedia Appendix 5.** Association of daily step count with home blood pressure in participants with 90 or more active days.

| Home BP | Participants | Model 1* | | | Model 2^†^ | | |
| --- | --- | --- | --- | --- | --- | --- | --- |
|  |  | β^‡^ (; mm Hg) | SE | P-value | β^‡^ (; mm Hg) | SE | P-value |
| Systolic BP | All participants  n=578 | -0.40 | 0.18 | 0.031 | 0.070 | 0.17 | 0.68 |
|  | Women  n=341 | -0.34 | 0.25 | 0.17 | 0.14 | 0.22 | 0.52 |
|  | Men  n=237 | -0.49 | 0.27 | 0.07 | -0.08 | 0.26 | 0.76 |
| Diastolic BP | All participants  n=578 | -0.35 | 0.13 | 0.008 | -0.04 | 0.12 | 0.76 |
|  | Women  n=341 | -0.45 | 0.17 | 0.01 | -0.13 | 0.15 | 0.36 |
|  | Men  n=237 | -0.23 | 0.21 | 0.27 | 0.07 | 0.21 | 0.75 |

*Model 1 was adjusted for age, sex, family structure, reported antihypertensive agent use, and watch wear time

^†^Model 2 was adjusted for model 1 covariates and body mass index.

^‡^β represents the change in BP (mmHg) for every 1,000 increase in daily steps
