# Supplementary material for: Association of Habitual Physical Activity With Home Blood Pressure in the Electronic Framingham Heart Study (eFHS): Cross-sectional Study
Source: J Med Internet Res. 2021 Jun 24;23(6):e25591. doi: 10.2196/25591 (PMC8277303; doi:10.2196/25591)
Supplement: Multimedia Appendix 6 [file jmir_v23i6e25591_app6.docx]

**Multimedia Appendix 6.** Association of daily step count with home blood pressure in participants with nine or more blood pressure readings.

| Home BP | Participants | Model 1* | | | Model 2^†^ | | |
| --- | --- | --- | --- | --- | --- | --- | --- |
|  |  | β^‡^ (; mm Hg) | SE | P-value | β^‡^ (; mm Hg) | SE | P-value |
| Systolic BP | All participants  n=540 | -0.54 | 0.17 | 0.002 | -0.059 | 0.16 | 0.71 |
|  | Women  n=326 | -0.42 | 0.23 | 0.07 | 0.13 | 0.20 | 0.51 |
|  | Men  n=214 | -0.68 | 0.25 | 0.01 | -0.33 | 0.25 | 0.19 |
| Diastolic BP | All participants  n=540 | -0.33 | 0.12 | 0.008 | 0.00 | 0.12 | 0.97 |
|  | Women  n=326 | -0.34 | 0.16 | 0.03 | 0.04 | 0.14 | 0.80 |
|  | Men  n=214 | -0.30 | 0.19 | 0.12 | -0.07 | 0.19 | 0.71 |

*Model 1 was adjusted for age, sex, family structure, reported antihypertensive agent use, and watch wear time

^†^Model 2 was adjusted for model 1 covariates and body mass index.

^‡^β represents the change in BP (mmHg) for every 1,000 increase in daily steps
