# Supplementary material for: Association of Habitual Physical Activity With Home Blood Pressure in the Electronic Framingham Heart Study (eFHS): Cross-sectional Study
Source: J Med Internet Res. 2021 Jun 24;23(6):e25591. doi: 10.2196/25591 (PMC8277303; doi:10.2196/25591)
Supplement: Multimedia Appendix 7 [file jmir_v23i6e25591_app7.docx]

**Multimedia Appendix 7.** Association of daily step count with home blood pressure using a threshold of 10 hours/day to define active days.

| Home BP | Participants | Model 1* | | | Model 2^†^ | | |
| --- | --- | --- | --- | --- | --- | --- | --- |
|  |  | β^‡^ (; mm Hg) | SE | P-value | β^‡^ (; mm Hg) | SE | P-value |
| Systolic BP | All participants n=634 | -0.45 | 0.17 | 0.007 | 0.010 | 0.15 | 0.95 |
|  | Women  n= 375 | -0.40 | 0.23 | 0.08 | 0.11 | 0.20 | 0.60 |
|  | Men  n= 259 | -0.51 | 0.23 | 0.03 | -0.17 | 0.23 | 0.45 |
| Diastolic BP | All participants n=634 | -0.33 | 0.12 | 0.006 | -0.03 | 0.11 | 0.81 |
|  | Women  n= 375 | -0.40 | 0.16 | 0.01 | -0.07 | 0.14 | 0.64 |
|  | Men  n= 259 | -0.25 | 0.18 | 0.17 | -0.03 | 0.18 | 0.87 |

*Model 1 was adjusted for age, sex, family structure, reported antihypertensive agent use, and watch wear time

^†^Model 2 was adjusted for model 1 covariates and body mass index.

^‡^β represents the change in BP (mmHg) for every 1,000 increase in daily steps
