# Supplementary figures and images for: Association of Habitual Physical Activity With Home Blood Pressure in the Electronic Framingham Heart Study (eFHS): Cross-sectional Study
Source: J Med Internet Res. 2021 Jun 24;23(6):e25591. doi: 10.2196/25591 (PMC8277303; doi:10.2196/25591)

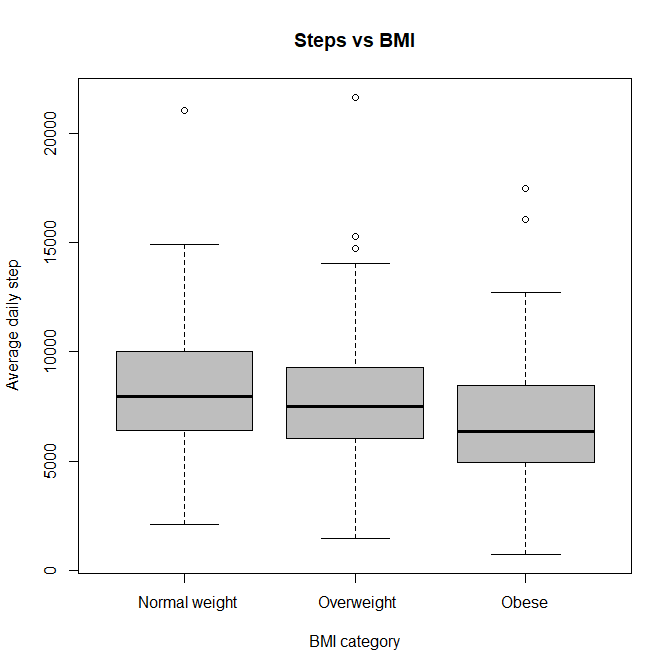

Supplement: Multimedia Appendix 8 [file jmir_v23i6e25591_app8.png]

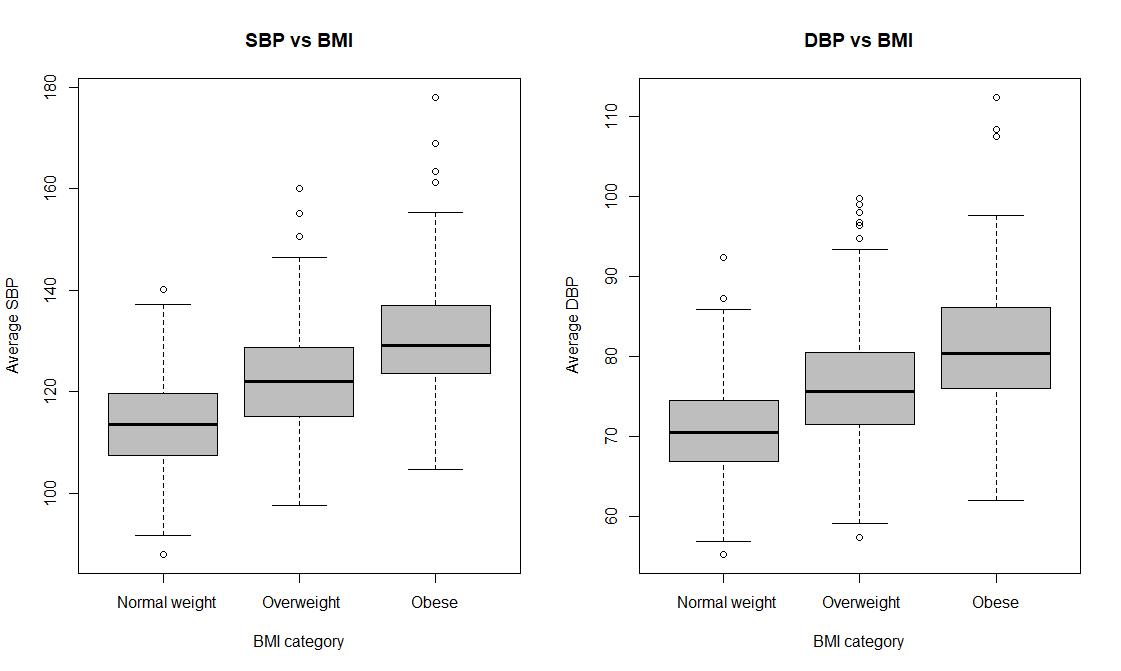

Supplement: Multimedia Appendix 9 [file jmir_v23i6e25591_app9.png]

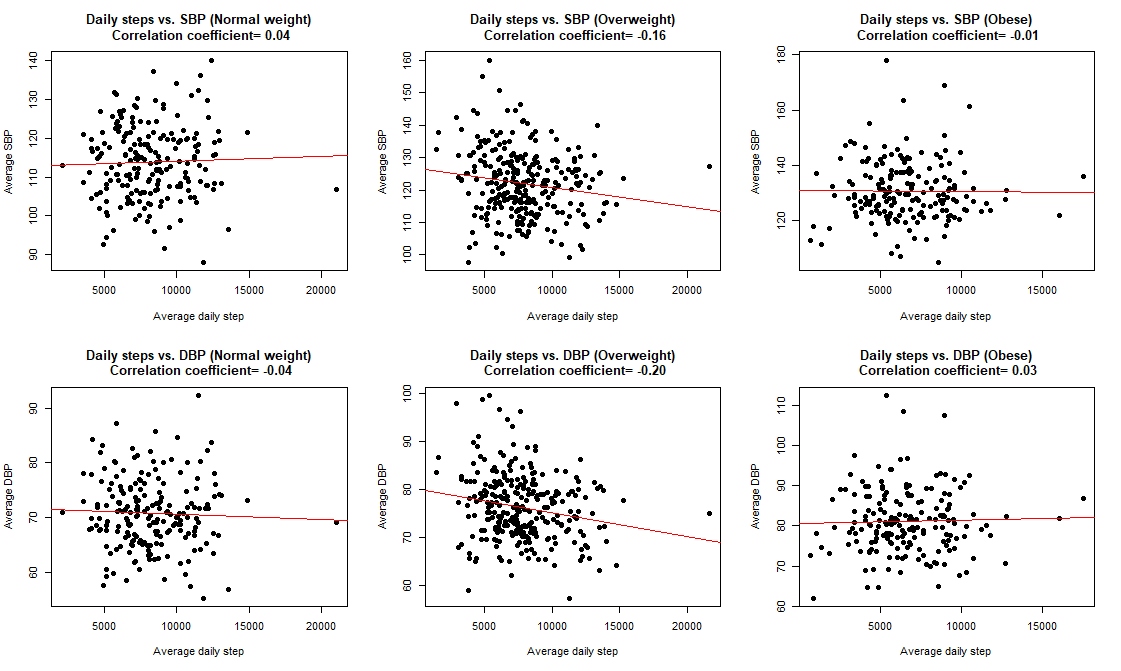

Supplement: Multimedia Appendix 10 [file jmir_v23i6e25591_app10.png]
