# Supplementary material for: Association of Habitual Physical Activity With Home Blood Pressure in the Electronic Framingham Heart Study (eFHS): Cross-sectional Study
Source: J Med Internet Res. 2021 Jun 24;23(6):e25591. doi: 10.2196/25591 (PMC8277303; doi:10.2196/25591)
Supplement: Multimedia Appendix 11 [file jmir_v23i6e25591_app11.docx]

**Multimedia Appendix 11.** Baseline characteristics of participants with and without a history of hypertension.

| Variable name | Participants with hypertension (n=183) | Participants without hypertension (n=477) | P value |
| --- | --- | --- | --- |
| Age | 57± 8 | 52± 9 | <0.05 |
| Women | 88 (48.1%) | 299 (62.7%) | <0.05 |
| Body Mass Index | 30.2± 5.3 | 26.8 ±4.7 | <0.05 |
| Number of active days | 406± 265 | 409± 269 | 0.95 |
| Average number of active hours | 13.2± 2.0 | 13.6 ±1.9 | 0.057 |
| Average daily steps | 6852± 2611 | 7879± 2708 | <0.05 |
