# Supplementary material for: Association of Habitual Physical Activity With Home Blood Pressure in the Electronic Framingham Heart Study (eFHS): Cross-sectional Study
Source: J Med Internet Res. 2021 Jun 24;23(6):e25591. doi: 10.2196/25591 (PMC8277303; doi:10.2196/25591)
Supplement: Multimedia Appendix 12 [file jmir_v23i6e25591_app12.docx]

**Multimedia Appendix 12.** Association of daily step count with home blood pressure in participants with a history of hypertension.

| Home BP | Model 1* | | | Model 2^†^ | | |
| --- | --- | --- | --- | --- | --- | --- |
|  | β^‡^ (; mm Hg) | SE | P-value | β^‡^ (; mm Hg) | SE | P-value |
| Systolic BP | -0.11 | 0.35 | 0.75 | 0.313 | 0.34 | 0.35 |
| Diastolic BP | -0.13 | 0.26 | 0.63 | 0.10 | 0.26 | 0.71 |

*Model 1 was adjusted for age, sex, family structure, reported antihypertensive agent use, and watch wear time. Sample size was 183 for this analysis

^†^Model 2 was adjusted for model 1 covariates and body mass index.

^‡^β represents the change in BP (mmHg) for every 1,000 increase in daily steps
