# Supplementary material for: Association of Habitual Physical Activity With Home Blood Pressure in the Electronic Framingham Heart Study (eFHS): Cross-sectional Study
Source: J Med Internet Res. 2021 Jun 24;23(6):e25591. doi: 10.2196/25591 (PMC8277303; doi:10.2196/25591)
Supplement: Multimedia Appendix 13 [file jmir_v23i6e25591_app13.docx]

**Multimedia Appendix 13.** Association of daily step count with home blood pressure tertile.

| Home BP | Participants | Model 1* | | | Model 2^†^ | | |
| --- | --- | --- | --- | --- | --- | --- | --- |
|  |  | β^‡^ (; mm Hg) | SE | P-value | β^‡^ (; mm Hg) | SE | P-value |
| Systolic BP^§^ | All participants n=660 | -0.04 | 0.01 | 0.0004 | -0.012 | 0.01 | 0.28 |
|  | Women  n= 387 | -0.04 | 0.02 | 0.02 | -0.01 | 0.02 | 0.62 |
|  | Men  n= 273 | -0.05 | 0.02 | 0.01 | -0.02 | 0.02 | 0.23 |
| Diastolic BP^\|\|^ | All participants n=660 | -0.03 | 0.01 | 0.021 | 0.0004 | 0.01 | 0.98 |
|  | Women  n= 387 | -0.04 | 0.02 | 0.02 | -0.01 | 0.02 | 0.65 |
|  | Men  n= 273 | -0.02 | 0.02 | 0.36 | 0.01 | 0.02 | 0.72 |

*Model 1 was adjusted for age, sex, family structure, reported antihypertensive agent use, and watch wear time

^†^Model 2 was adjusted for model 1 covariates and body mass index.

^‡^β represents the change in BP tertile for every 1,000 increase in daily steps

^§^Systolic BP tertile cutoffs were <116 mm Hg (tertile 1), 116-127 mm Hg (tertile 2), and >127 mm Hg (tertile 3).

^||^Diastolic BP tertile cutoffs were <72 mm Hg (tertile 1), 72-79 mm Hg (tertile 2), and >79 mm Hg (tertile 3).
